# Is Surveillance Colonoscopy Necessary for Patients with Sporadic Gastric Hyperplastic Polyps?

**DOI:** 10.1371/journal.pone.0122996

**Published:** 2015-04-13

**Authors:** Hailong Cao, Nana He, Shuli Song, Mengque Xu, Meiyu Piao, Fang Yan, Bangmao Wang

**Affiliations:** 1 Department of Gastroenterology and Hepatology, General Hospital, Tianjin Medical University, Tianjin, China; 2 Division of Gastroenterology, Hepatology and Nutrition, Department of Pediatrics, Vanderbilt University Medical Center, Nashville, Tennessee, United States of America; Queen Mary Hospital, HONG KONG

## Abstract

**Background:**

Gastric polyps, such as adenomas and hyperplastic polyps, can be found in various colonic polyposis syndromes. Unlike in sporadic gastric adenomas, in which the increased risk of colorectal neoplasia has been well characterized, information in sporadic gastric hyperplastic polyps was limited.

**Aim:**

To evaluate the association of sporadic gastric hyperplastic polyps with synchronous colorectal neoplasia in a large cohort.

**Methods:**

Patients with sporadic gastric hyperplastic polyps who underwent colonoscopy simultaneously or within six months were consecutively enrolled. Each patient was compared with two randomly selected age and sex matched controls without gastric polyps who also underwent colonoscopy in the same period. Data of patients’ demographics and characteristics of the gastrointestinal polyps were documented.

**Results:**

A total of 261 cases in 118,576 patients who underwent esophagogastroduodenoscopy were diagnosed as sporadic gastric hyperplastic polyps, and 192 of 261 (73.6%) patients underwent colonoscopy. Colorectal neoplasias were identified in 46 (24.0%) of 192 cases and in 40 (10.4%) of 384 controls (*P*＜0.001). The mean size and distribution of colorectal neoplasias were not significantly different between the two groups. There was a significantly higher rate of colorectal adenoma (*odds ratio* [*OR*] 3.2, 95% *confidence interval* [*CI*] 1.9–5.3) in the gastric hyperplastic polyps group than in the control group, while the prevalence of colorectal cancer was similar in the two groups. Logistic regression analysis also suggested that the presence of gastric hyperplastic polyps (*OR* 2.5, 95% *CI* 1.5–4.0) was an independent risk factor for colorectal neoplasias.

**Conclusion:**

The risk of colorectal adenoma increases in patients with sporadic gastric hyperplastic polyps, and surveillance colonoscopy for these patients should be considered.

## Introduction

Gastric polyps are sessile or pedunculated lesions that originate in the gastric epithelium or submucosa and protrude into the stomach lumen. Gastric polyps can be found in approximately 1–6% of esophagogastroduodenoscopy (EGD) examination.[1–3] Gastric polyps, such as adenomas and hyperplastic polyps, can be found in various colonic polyposis syndromes, such as familial adenomatous polyposis,[4] Peutz-Jeghers syndrome,[5] and Cronkhite-Canada syndrome.[6] Recent studies have shown that patients with sporadic gastrointestinal adenomas or cancers also have a significantly greater incidence of colonic neoplasias.[7–11] However, until now information in patients with sporadic gastric hyperplastic polyps was limited.

Gastric hyperplastic polyp is one of the common types of epithelial polyps with the relative prevalence ranging from 17.0%-75.6%.[12–16] It is traditionally assumed to be a benign lesion,[15,16] however, it may have malignant transformation potential similar to the adenoma.[17–19] An interesting meta-analysis was recently performed to evaluate the risk of colorectal neoplasias in patients with upper gastrointestinal polyps.[20] Remarkably, the prevalence of colorectal polyps was higher in patients with gastric polyps than in those without gastric polyps. However, most of the data were collected from gastric fundic gland polyps and adenomas, gastric hyperplastic polyps were rarely included. Thus, further studies are necessary to verify the relationship between gastric hyperplastic polyps and colorectal neoplasia. This is of particular concern, as this notion may lead to a different management strategy. To address this issue and also provide more evidence for the management of gastric hyperplastic polyps in clinical practice, we evaluated the relationship between gastric hyperplastic polyps and synchronous colorectal neoplasia in a relatively large population.

## Methods

### Materials

All consecutive patients who underwent routine EGD at the Digestive Endoscopy Center of General Hospital, Tianjin Medical University between January 2011 and December 2013 were prospectively recruited. The indications for EGD were due to various symptoms, such as dyspepsia, abdominal pain, and gastroesophageal reflux. Other kinds of endoscopies such as emergent and therapeutic EGD were not included. When gastric hyperplastic polyps were histologically confirmed, patients were prospectively required to undergo colonoscopy simultaneously or within six months. All written informed consents for both EGD and colonoscopy were granted from the patients before the procedure, and ethical committee approval was obtained from Ethics Committee of General Hospital, Tianjin Medical University. Data including patients’ age, sex, the indications of endoscopy, *H*. *pylori* infection, and the number and histology of gastric polyps and colonic neoplasias were documented. *H*. *pylori* infection was considered to be current if at least one of the following tests showed a positive result: (1) rapid urease test; (2) urea breath test; (3) endoscopic gastric mucosal biopsy. According to the US Surveillance of Colorectal Polyp Resection Guidelines of 2006,[21] high risk adenomas were defined as adenomas with diameters of ≥1 cm, adenoma with a villous component, adenoma with high grade dysplasia (HGD), or three or more adenomas. Patients with multiple colorectal neoplasia were categorized according to the most advanced lesion. Two independent researchers extracted data separately, and both researchers reviewed every case. Then mutual agreement was reached through discussion when there were inconsistencies.

To further exploit the clinical implications of our identified rate of colorectal neoplasia in patients with gastric hyperplastic polyps, we collected symptomatic patients who did not show any gastric polyps and underwent both EGD and colonoscopy as controls, and then analyzed the differences between each study case with two randomly selected age and sex matched controls. Moreover, cases and controls had similar indications for the endoscopy.

Exclusion criteria were as follows: 1) Patients with other co-existing pathologic types of polyps in the stomach. 2) Patients with gastric malignancies or submucosa tumors such as adenocarcinoma, carcinoid tumor, malignant lymphoma or MALToma. 3) Patients with any kinds of polyposis syndromes. 4) Patients with a history of intestinal cancer or inflammatory bowel disease, 5) Patients with a history of colonic resection.

### Endoscopic procedure and pathological evaluation

According to the protocol, after gastric hyperplastic polyps were confirmed, patients were asked to undergo colonoscopy within six months. Two experienced independent endoscopists performed colonoscopy carefully during insertion and withdrawal under anesthesia, and they didn’t know whether the patients had gastric polyps before the procedure. Electronic endoscopes (Olympus CF-Q260, Olympus Optical Co., Tokyo, Japan) were used for all procedures. Patients were prescribed polyethylene glycol lavage (PO) for bowel preparation. Patients were orally lavaged, and watery diarrhea excretion prior to the procedure indicated adequate intestinal preparation. Pathological evaluations were performed by two certified pathologists according to standard pathology laboratory protocols. All collected specimens were fixed in 10% formalin within 1 h of removal and then fixed for a minimum of 4 h. Hematoxylin and eosin staining was used for histopathological evaluation and classification.

### Statistical analysis

All statistical analyses were performed using SPSS 17.0 (Chicago, IL, USA) for Windows. Risks of colorectal neoplasia between gastric hyperplastic polyps and controls were compared by χ^2^ test or Fisher exact test. Means and standard deviation (SD) were calculated for continuous variables and were compared with the Student’s *t*-test. If the continuous variables were not normally distributed, then medians/interquartile range and a Mann-Whitney-*U* test would be used. Logistic regression analysis was used to evaluate the *odds ratios* (*OR*) and 95% *confidence intervals* (*CI*) for colorectal neoplasia. Age (≥50 years), sex, aspirin medications, history of diabetes mellitus, and body mass index (>25 kg/m^2^) were selected as possible confounding factors. The level of statistically significance was set at two-tailed *P* < 0.05.

## Results

### Basic clinical features

Among a total of 118,576 consecutive patients who underwent EGD, the mean age was 49.3 years old (range: 7–98), 48.3% were males, and 51.7% were females. A total of 261 gastric hyperplastic polyps were diagnosed, and 73.6% of patients (192/261) underwent a colonoscopy simultaneously or within six months. The mean age of these 192 patients with gastric hyperplastic polyps was 58.0 years old (range: 18–84), and the male-female ratio was 66: 126. The mean size was 5.0±6.0 mm (median ± quartile interval), with a range of 1 to 50 mm.

The majority of cases (60.9%, 117/192) had one hyperplastic polyp. Histological examinations revealed gastric hyperplastic polyps with HGD in 1 case (0.5%), and LGD in 50 cases (26.0%). Gastric hyperplastic polyps were frequently located in the corpus (28.9%), antrum (27.0%) and fundus (22.3%), respectively (Table 1).

**Table 1:** Characteristics of the 192 patients with gastric hyperplastic polyps.

Parameters	
**Sex, n(%)**	
Male	66(34.4)
Female	126(65.6)
**Age(mean ± SD)**	58.0±13.1
≥50	151(78.6)
<50	41(21.4)
**Family history of gastric cancer, n(%)**	16(8.3)
**Number of gastric hyperplastic polyps, n(%)**	
Single	117(60.9)
Multiple	75(39.1)
**Size of polyps (median ± quartile interval)**	5.0±6.0
**Distribution, n(%)** *	
Cardia	36(17.1)
Fundus	47(22.3)
Corpus	61(28.9)
Angle	4(1.9)
Antrum	57(27.0)
Pylorus	6(2.8)
**Pathology, n(%)**	
with low grade dysplasia	50(26.0)
with high grade dysplasia	1(0.5)
without dysplasia	141(73.5)

* The number of the patients with gastric hyperplastic polyps. One patient might have more than one polyp located in different regions.

Each case with two age and sex matched patients presenting to our center for both EGD and colonoscopy were randomly selected as the control group. *H*. *pylori* infection was detected in 51.1% (95/186, non responder 6) of patients with gastric hyperplastic polyps, and 42.9% (160/373, non responder 11) in the control group, respectively, however a statistically significant decrease was not found (*P* = 0.070). Mean body mass index was not significantly different between the two groups.

### Colorectal neoplasia in patients with gastric hyperplastic polyps

The number of patients with family histories of colorectal cancer and personal histories of colorectal adenomas was not significantly different between the gastric hyperplastic polyps group and the control group. A history of aspirin medication was more prevalent in the gastric hyperplastic group. History of smoking, alcohol drinking, diabetes mellitus, and mean body mass index were not significantly different between the two groups (Table 2).

**Table 2:** Clinical characteristics of the gastric hyperplastic polyps group and the control group.

Parameters	Gastric hyperplastic polyps group(n = 192, %)	Control group (n = 384, %)	*OR (95% CI)*	*P value*
**Family history of colorectal cancer**				
Yes	9(4.7)	20(5.2)	0.897(0.400–2.012)	0.793
Non responder	6(3.1)	11(2.9)		
**Personal history of colorectal adenomas**				
Yes	35(18.2)	51(13.3)	1.456(0.910–2.329)	0.116
Non responder	6(3.1)	11(2.9)		
**History of smoking**				
Yes	35(18.2)	56(14.6)	1.306(0.822–2.075)	0.258
Non responder	6(3.1)	11(2.9)		
**History of alcohol drinking**				
Yes	23(12.0)	37(9.6)	1.323(0.761–2.300)	0.319
Non responder	6(3.1)	11(2.9)		
**History of diabetes mellitus**				
Yes	20(10.4)	45(11.7)	0.878(0.502–1.536)	0.649
Non responder	6(3.1)	11(2.9)		
**Aspirin medication**				
Yes	35(18.2)	46(12.0)	1.638(1.015–2.644)	0.042
Non responder	6(3.1)	11(2.9)		
**Infection of *Helicobacter Pylori***				
Positive	95(49.5)	160(41.7)	1.390(0.976–1.978)	0.067
Non responder	6(3.1)	11(2.9)		
**Body mass index, kg/m** ^**2**^ **±SD**	24.0±2.8	23.8±1.6		0.393

Overall, colorectal neoplasia including colorectal adenoma (CRA) and cancer (CRC) was detected in 46 (42 CRA and 4 CRC) of the 192 subjects (24.0%) enrolled in the gastric hyperplastic polyps group, and in 40 (31 CRA and 9 CRC) of the 384 subjects (10.4%) in control group (*odds ratio* [*OR*], 2.7; 95% *confidence interval* [*CI*], 1.7–4.3, *P*＜0.001). The mean size (median ± quartile interval: 5.5±6.8 mm *vs* 7.0±26.0 mm, *P* = 0.340) and distribution of colorectal neoplasia were not significantly different. There was a significantly higher rate of colorectal adenomas (21.9% *vs* 8.1%, *OR* 3.2, 95% *CI* 1.9–5.3) in the patients with gastric hyperplastic polyps compared to that in the control group, while the rate of colorectal cancers was similar in both groups (Table 3). Moreover, colorectal high risk adenomas were detected in 27 of the 192 subjects (14.1%) in the gastric hyperplastic polyps group, and in 15 of the 384 subjects (3.9%) in control group. The prevalence of all subtypes of high risk adenomas was also significantly higher in the gastric hyperplastic polyps group than in the control group (*P*＜0.001), as listed in Table 4.

**Table 3:** Prevalence of colorectal neoplasia in the gastric hyperplastic polyps group and the control group.

	Gastric hyperplasticpolyps group	Control group	*OR*(95%*CI*)	*P* value
All colorectal neoplasia, n. (%)	46(24.0)	40(10.4)	2.710(1.701–4.317)	0.000
Colorectal adenoma, n. (%)	42(21.9)	31(8.1)	3.188(1.930–5.267)	0.000
Colorectal cancer, n. (%)	4(2.1)	9(2.3)	0.887(0.269–2.916)	1.000
Colorectal advanced adenoma, n. (%)	27(14.1)	15(3.9)	4.025(2.086–7.768)	0.000
Colorectal non-advanced adenoma, n. (%)	15(7.8)	16(4.2)	1.949(0.942–4.032)	0.068

**Table 4:** Characteristics of colorectal neoplasia in the gastric hyperplastic polyps group and the control group.

Parameters	Gastric hyperplastic polyps group(n = 46)	Control group(n = 40)	*OR* (95% *CI*)	*P* value
**Number of colorectal neoplasia**				
Single	28	30	2.015(1.166–3.482)	0.013
Multiple	18	10	3.869(1.749–8.556)	0.001
**Distribution, n(%)**				
^†^Proximal	19(41.3)	17(42.5)	2.371(1.203–4.675)	0.016
^‡^Distal	18(39.1)	16(40.0)	2.379(1.185–4.778)	0.012
Proximal and distal	9(19.6)	7(17.5)	2.649(0.971–7.224)	0.049
**Number of colorectal high risk adenoma** *	27	15	4.025(2.086–7.768)	0.000
Large ≥ 1 cm	9	3	6.246(1.671–23.346)	0.002
Tubulovillous/villous adenoma	14	8	3.697(1.523–8.973)	0.002
High grade dysplasia	5	2	5.107(0.982–26.569)	0.044
Number ≥ 3	12	5	5.053(1.754–14.560)	0.001

^†^Proximal: cecum, ascending colon, hepatic flexure, transverse colon

^‡^Distal: splenic flexure, descending colon, sigmoid colon, rectum.

* The number of the patients with colorectal high risk adenomas.

One high risk adenoma might have more than one characteristic of the following: large ≥1 cm, adenoma with a villous component, adenoma with high grade dysplasia, or three or more adenomas.

### Multivariate logistic regression analysis

Multivariate logistic regression analysis showed that the presence of gastric hyperplastic polyps (*OR*, 2.5; 95% *CI*, 1.5–4.0) was an independent risk factor associated with the higher prevalence of colorectal neoplasia (Table 5).

**Table 5:** Logistic regression analysis for colorectal neoplasia in the gastric hyperplastic polyps group and the control group.

Parameters	*OR*	95% *CI*	*P* value
Presence of gastric hyperplastic polyps	2.489	1.546–4.007	0.000
Age ≥ 50 years	1.628	0.891–2.974	0.113
Male	1.181	0.698–1.999	0.535
Aspirin medication	0.777	0.376–1.606	0.495
History of diabetes mellitus	1.199	0.576–2.495	0.628
Body mass index >25 kg/m^2^	1.208	0.691–2.112	0.507

## Discussion

Gastric polyps can be easily found during the endoscopy procedures, however, the management remains a challenge for the endoscopists.[1,22] It has been reported that gastric adenomatous polyps increase the risk of colorectal neoplasia,[7,8] therefore endoscopists should put more emphasis on colonoscopy screening rather than only biopsy or polypectomy. However, there are few studies concerning the prevalence of colorectal neoplasia in patients with gastric hyperplastic polyps. Cappell *et al*. reported a significantly greater incidence of colonic polyps in patients with nonmalignant gastric polyps including only 14 patients with hyperplastic polyps, 20 inflammatory polyps, and 7 adenomas.[8] Only patients with gastric hyperplastic polyps were enrolled in our study. Age-matched and sex-matched controls were randomly selected to minimize the potential for selection bias. The present study provides current information on the relationships between gastric hyperplastic polyps and synchronous colorectal neoplasia. Based on our current data, there was a significantly higher rate of colorectal adenoma in the gastric hyperplastic polyps group than in the control group, thus, the management of these patients should also focus on the colonoscopy screening.

Several strengths are included in the present study. Firstly, the study adopted a simple and readily accepted method (EGD and colonoscopy), so the risk of missing polyps was greatly reduced compared to gastroenterography. Secondly, we chose a recent period to minimize the influence of confounding factors such as changes in clinical practice and histological diagnosis. Thirdly, although it is not a population-based screening study, the present study with a relatively large sample size is based on the results of EGD for more than 100 000 consecutive patients. Moreover, as this study was conducted in a tertiary endoscopic center, the possibility of clinical heterogeneity is also minimized.

However, several limitations should also be mentioned. Firstly, a small portion of the patients (26.4%) with gastric hyperplastic polyps in the present study did not undergo colonoscopy possibly due to lack of clinical compliance and other unknown reasons. We also compared the characteristics of these additional 69 patients with those in study group. We found that the mean age, sex and the indications for the endoscopy of these patients with gastric hyperplastic polyps who didn't receive a colonoscopy was similar to those in study group(S1 Table). Secondly, this study was conducted in a tertiary endoscopic center, so selection bias may not be ignored. In addition, other potential confounding factors for example a history of hyperlipidemia were not investigated. We collected information of body mass index, however, no significant difference was shown between the two groups.

The rate of colorectal neoplasia in the control patients in the present study was 10.4%, which was consistent with that of 10.7% in symptomatic patients according to a previous colonoscopy database study in China. [23] However, the frequencies of colorectal neoplasia in Western populations ranged from 20.4% to 37.5%, [24–26] which was higher than our population. The geographical differences and age of the patients may play a vital role on the different prevalence in Western and Eastern areas.

The reasons why patients with gastric hyperplastic polyps are more prone to have colorectal neoplasias are unknown. Some studies suggested that the types and frequencies of genetic alterations occurring in gastric and colorectal polyps are similar. They may refer to alterations in *Apc*, *K-RAS*, *p53* genes and microsatellite instability.[27] Overexpression of *FAT10* is the characteristic of numerous types of carcinoma including gastric and colon carcinomas.[28] To our knowledge, only one study has reported three of four cases of hyperplastic gastric polyposis (presence of multiple gastric hyperplastic polyps) had colonic carcinomas which was possibly due to hypergastrinaemia.[29] However, Lahner *et al*. recently showed that atrophic gastritis-related hypergastrinemia was not associated with high risk of colorectal neoplasia.[30] In addition, conflicting data have shown the association between *H*. *pylori* infection and colorectal neoplasia.[31–35] In the present study, *H*. *pylori* infection rate was similar in the patients with gastric hyperplastic polyps and the control group. Thus, our findings cannot be well explained by the factor of *H*. *pylori* infection. Further studies on the detailed mechanisms remain to be done.

In conclusion, the risk of colorectal adenoma increases in patients with sporadic gastric hyperplastic polyps, and surveillance colonoscopy for these patients should be considered. Further studies are required to further validate these results and investigate the reasons, and to confirm whether it will lead to a new strategy for the management of gastric hyperplastic polyps.

## Supporting Information

S1 Table Indications for the procedures in both case and control groups. (DOCX)
