# Supplementary material for: Is Surveillance Colonoscopy Necessary for Patients with Sporadic Gastric Hyperplastic Polyps?
Source: PLoS One. 2015 Apr 13;10(4):e0122996. doi: 10.1371/journal.pone.0122996 (PMC4395217; doi:10.1371/journal.pone.0122996)
Supplement: S1 Table — (DOCX) [file pone.0122996.s001.docx]

Supplementary Table Indications for the procedures in both case and control groups

| **Indications for EGD** | **Gastric hyperplastic**  **polyps group** | **Control group** | ***P* value** |
| --- | --- | --- | --- |
| Dyspepsia | 45(23.4) | 135(35.16) | ＞0.05 |
| Abdominal pain | 49(25.5) | 98(25.52) | ＞0.05 |
| Gastroesophageal reflux | 42(21.9) | 71(18.49) | ＞0.05 |
| Others* | 56(29.2) | 80(21.35) | ＞0.05 |

Others: patients with indications of melena, hematochezia, anemia, fatigue, anorexia, malnutrition and so on.
